# A Novel *KCNA1* Variant in a Patient With Tremor and Autism Spectrum Disorder Causes Mixed LOF/GOF Defects of Kv1.1 Channels

**DOI:** 10.1002/acn3.70536

**Published:** 2026-09-21

**Authors:** Juan Darío Ortigoza‐Escobar, Laura Marti‐Sánchez, Loreto Martorell, Giorgia Dinoi, Antonio Vittorio Buono, Annamaria De Luca, Antonella Liantonio, Paola Imbrici

**Affiliations:** ^1^ Movement Disorders Unit, Pediatric Neurology Department Institut de Recerca Hospital Sant Joan de Déu Barcelona Barcelona Spain; ^2^ U‐703 Centre for Biomedical Research on Rare Diseases (CIBER‐ER) Instituto de Salud Carlos III Barcelona Spain; ^3^ European Reference Network for Rare Neurological Diseases (ERN‐RND) Barcelona Spain; ^4^ Unitat de Genòmica‐NGS, Servei Diagnòstic de Laboratori, Hospital Sant Joan de Déu Barcelona Spain; ^5^ Department of Pharmacy‐Drug Sciences University of Bari “Aldo Moro” Bari Italy

**Keywords:** autism spectrum disorders, *KCNA1*, Kv1.1, patch clamp, tremor, voltage clamp

## Abstract

Variants in *KCNA1*, encoding the Kv1.1 potassium channel, cause neurological disorders including episodic ataxia and developmental and epileptic encephalopathy. We identified a novel *KCNA1* variant (A401T) in a 16‐year‐old patient with autism spectrum disorder, borderline intellectual disability, and tremor, without episodic ataxia or epilepsy. Functional analysis in HEK293 cells showed that A401T markedly reduced potassium currents, slowed activation, and accelerated C‐type inactivation, consistent with loss‐of‐function (LOF), while shifting channel activation to more negative potentials, indicating gain‐of‐function (GOF). Overall, our findings expand both the clinical and functional spectrum of *KCNA1*‐related disorders by identifying autism spectrum disorder and tremor associated with a mixed LOF/GOF *KCNA1* variant.

## 1 Introduction

The voltage‐gated potassium channel Kv1.1, encoded by *KCNA1*, is widely expressed in the central and peripheral nervous systems, where it contributes to action potential repolarization and regulates neuronal excitability and neurotransmitter release [1]. Loss‐of‐function (LOF) *KCNA1* variants are classically associated with episodic ataxia Type 1, a rare cerebellar disorder characterized by recurrent attacks of ataxia and myokymia beginning in childhood [2]. However, advances in clinical and genetic studies have broadened the phenotypic spectrum to include epilepsy, developmental and epileptic encephalopathy, migraine, paroxysmal dyskinesia, hereditary spastic paraplegia, nystagmus, cataplexy, and metabolic or electrolyte abnormalities reported in isolated cases, including hypomagnesemia (Figure S1) [3, 4, 5, 6]. Only two gain‐of‐function (GOF) variants have been reported to date, both associated with epilepsy [7, 8]. *KCNA1*‐related disorders show marked phenotypic heterogeneity, even among carriers of the same variant, suggesting variable expressivity and incomplete penetrance. In the differential diagnosis of episodic ataxia, other genetic conditions should be considered, particularly *CACNA1A*‐related episodic ataxia Type 2 and biallelic *PRRT2*‐associated disorders, among other paroxysmal channelopathies [9, 10, 11]. Although epilepsy and ataxia remain the most recognized manifestations, increasing evidence indicates that variants in voltage‐gated potassium channels may also contribute to neurodevelopmental phenotypes such as autism spectrum disorder and intellectual disability [12]. However, the role of *KCNA1* variants in isolated neurodevelopmental presentations without epilepsy is still poorly defined.

Kv1.1 channel assembles as a tetramer of four α subunits, each containing six transmembrane helices that form the voltage‐sensing domain (S1–S4) and the pore domain (S5–S6), both essential for channel function [13, 14]. In this study, we describe a novel missense variant, A401T, located in the S6 segment and close to the Pro‐Val‐Pro (PVP) motif of Kv1.1 in a 16‐year‐old boy presenting with autism spectrum disorder and tremor in the absence of epilepsy. We hypothesized that this S6 variant might alter channel gating in a manner distinct from previously described *KCNA1* mutations and contribute to a non‐epileptic neurodevelopmental phenotype. Using patch‐clamp electrophysiology, we investigated the functional consequences of this amino acid substitution and demonstrated a mixed LOF/GOF molecular profile for the A401T variant. See Supporting Information for methods description [15, 16, 17].

## 2 Case Study

A 16‐year‐old male with mild autism spectrum disorder and borderline intellectual functioning (total IQ, 79) was evaluated for a distal postural and action tremor. Pregnancy was appropriately monitored and uncomplicated. He was born at term at home because the family did not reach the hospital in time and was subsequently transferred to the hospital, without documented neonatal complications. Motor development was normal, with independent walking at approximately 12 months and no developmental regression. Language acquisition was delayed: Before three years of age, his vocabulary was largely limited to “mother” and “father”; additional single words emerged at approximately three years of age, followed shortly thereafter by simple sentences. He received outpatient speech and language therapy and educational support. He was referred for a specialized autism assessment at 8 years of age because of difficulties in social interaction, reduced eye contact, and impaired verbal expression, and autism spectrum disorder was diagnosed at 10 years of age. He had transient difficulties initiating sleep during early childhood, which subsequently resolved, and currently has no clinically relevant sleep disorder. There was no known family history of tremor, episodic ataxia, epilepsy, myokymia, muscle cramps, autism spectrum disorder, intellectual disability, hypomagnesemia, or another neurological or neurodevelopmental disorder. His siblings were reportedly healthy, and there was no apparent consanguinity.

The tremor was first noticed by the patient and his mother approximately three to four years before the most recent clinical assessment. It predominantly affected the hands and occasionally the feet, was mild and non‐disabling, and had improved since its onset. There was no history of early‐onset movement abnormalities, episodic unsteadiness, falls, paroxysmal ataxia, involuntary jerks, abnormal posturing, painful cramps, or other movement disorders. Neurological examination showed normal tone, strength, coordination, and gait, without episodic ataxia, dysarthria, nystagmus, or clinically apparent myokymia. Deep tendon reflexes were normal and symmetric, plantar responses were flexor bilaterally, and there was no clonus, spasticity, fasciculation, weakness, or muscle atrophy. Facial and limb myokymia were specifically sought, with no visible muscle rippling, fasciculations, or continuous muscle activity. The Archimedes spiral was not significantly impaired. The patient reported brief subjective episodes of hand stiffness while holding a pen for prolonged periods. Direct examination during handwriting showed no abnormal posture, involuntary flexion or extension of the fingers or wrist, or other dystonic features. The phenomenon was not captured on video and could not be objectively classified as dystonia or myotonia.

Electromyography of the right brachioradialis muscle showed no myokymic discharges, neuromyotonic activity, or repetitive discharges. Brain MRI performed at 13 years of age was specifically re‐evaluated by a radiologist and showed normal cerebral and cerebellar morphology and volume, without evidence of cerebellar atrophy (Figure S2). No EEG was performed because the patient had no clinical seizures or other events suggestive of epilepsy. No metabolic or electrolyte abnormalities were documented. At the most recent follow‐up, at 17 years and 3 months of age, growth parameters were within normal limits for age and sex: Height 180 cm, weight 73.3 kg, body mass index 22.62 kg/m^2^, and occipitofrontal circumference 57 cm. Previous longitudinal growth had also been normal. Because the tremor was mild, improving, and non‐disabling, no pharmacological treatment was initiated, including acetazolamide, other carbonic anhydrase inhibitors, 4‐aminopyridine, or other tremor‐directed treatments.

Genetic analysis identified the heterozygous *KCNA1* variant NM_000217.3:c.1201G > A, p.(Ala401Thr), classified as likely pathogenic according to the ACMG and ACGS guidelines (PP3‐moderate, PM1, PM2‐supporting, PP2‐supporting, and PS3‐supporting) [18, 19]. Whole‐exome sequencing did not identify another potentially relevant variant that could explain the neurodevelopmental phenotype. The *KCNA1* variant was absent in the mother. Paternal segregation analysis was not possible because the father had died approximately seven years earlier and no DNA sample was available. The father was reportedly clinically healthy, but his medical records were unavailable. Therefore, a *de novo* occurrence cannot be confirmed, and paternal inheritance cannot be definitively excluded.

The Ala401Thr variant, named A401T, is positioned within the S6 transmembrane helix of the Kv1.1 channel, near the conserved PVP motif. Sequence alignment revealed that this residue is strongly conserved across Kv1 family members (Figure 1A,B). Because the patient carries the mutation in the heterozygous state, functional Kv1.1 channels are expected to consist of a combination of wild‐type (WT) and A401T mutant subunits. *In silico* prediction tools suggested a LOF effect (https://cbosselmann.shinyapps.io/prefeKt/).

**FIGURE 1:**
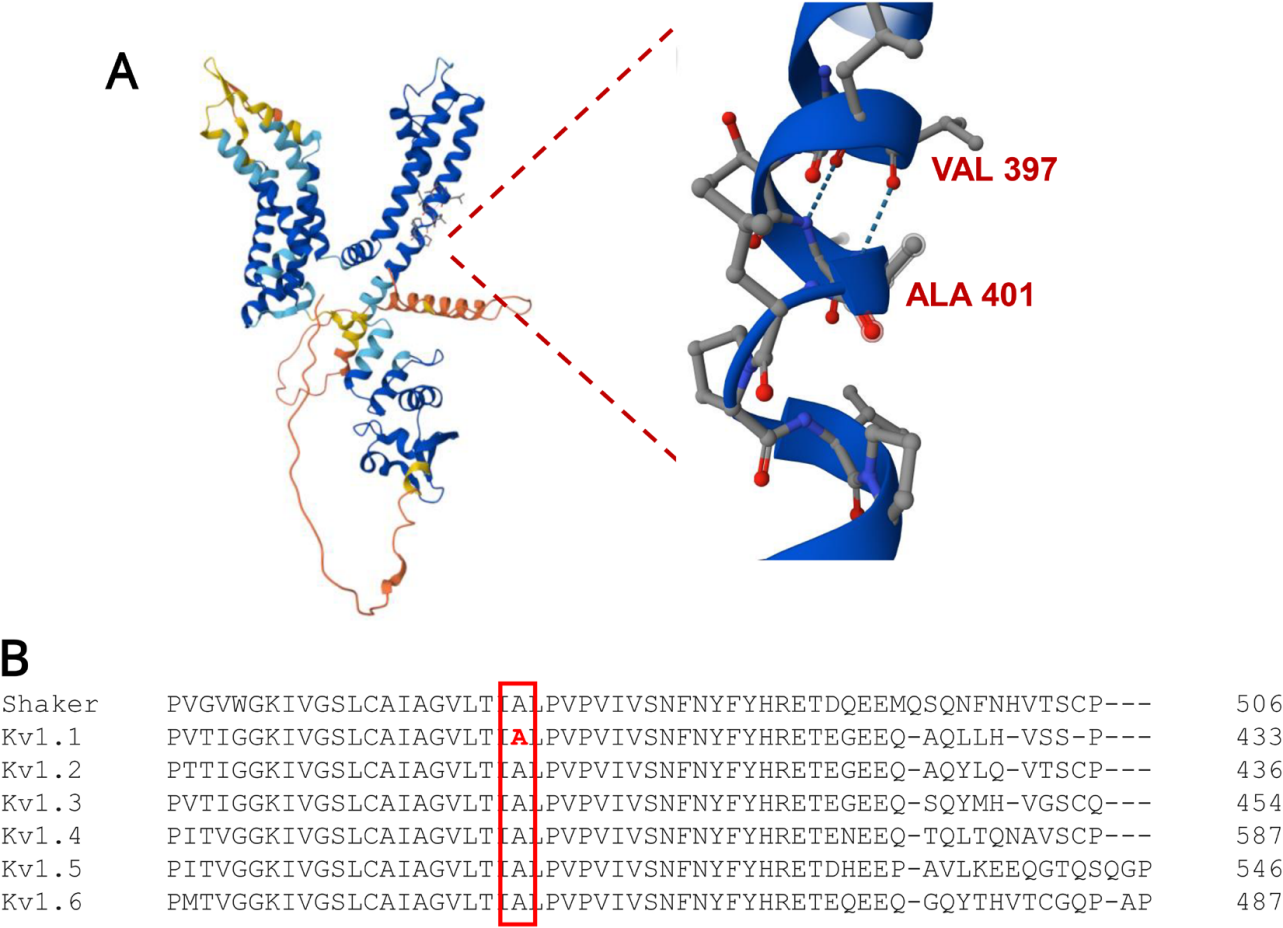
(A) Left: AlphaFold‐predicted structure of the alpha subunit of the Kv1.1 channel. Right: Enlarged view of the pore‐forming S6 transmembrane segment showing the hydrogen‐bonding interaction between Ala401 and Val397 within the S6 helix. (B) Amino acid alignment of Kv1 channels.

To assess whether the A401T substitution disrupts Kv1.1 channel function and could underlie the clinical manifestations of autism and tremor observed in the patient, HEK293 cells were transiently transfected with cDNAs encoding Kv1.1 WT or A401T alone (7 μg each), or co‐transfected with equal amounts of WT and mutant constructs (3.5 μg each), and currents were recorded using whole‐cell patch‐clamp. We then compared the amplitude and biophysical characteristics of currents generated by A401T homomeric channels and by heteromeric WT + A401T channels with those produced by WT channels. Across all test voltages, A401T‐expressing cells generated current amplitudes approximately 10‐fold lower than WT (Figure 2A–D; Table S1). Similarly, co‐expression of WT and A401T subunits resulted in current amplitudes approximately 40% of WT values (0.84 ± 0.13 nA vs. 2.10 ± 0.20 nA; Figure 2D; Table S1). To evaluate the impact of the A401T mutation on voltage‐dependent activation, tail currents recorded at −50 mV were analyzed to build activation curves (Figure 2E; Table S1). Compared with WT channels, A401T and WT + A401T channels exhibited a significant hyperpolarizing shift of approximately −12 mV and −14 mV, respectively, in the voltage dependence of activation, relative to WT (Figure 2E; Table S1). We next examined whether the A401T variant altered channel gating kinetics. A401T channels activated more slowly than WT channels at all tested voltages, compared with WT (Figure 2F; Table S1). In contrast, deactivation kinetics of A401T channels were comparable to those of WT channels (Figure 2G; Table S1). Finally, both A401T and WT + A401T channels exhibited slightly accelerated C‐type inactivation, whereas recovery from inactivation was not significantly different from that of WT channels (Figure 2H,I; Table S1).

**FIGURE 2:**
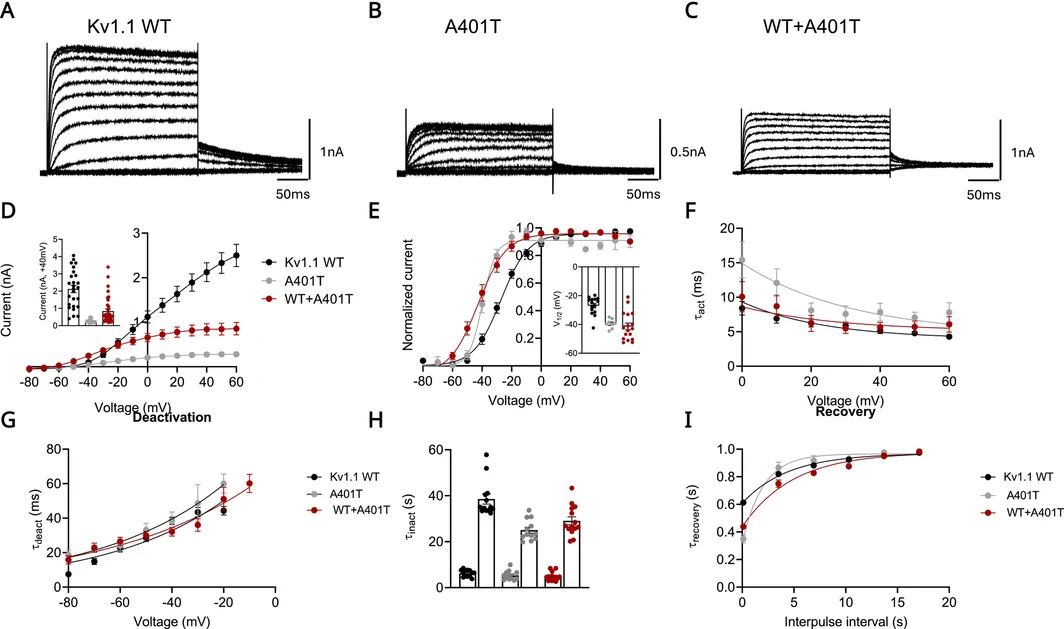
(A–C) Representative current traces evoked by 200 ms depolarizing steps from a holding potential of −80 to +60 mV from Kv1.1 WT (A), A401T (B), and WT + A401T (C) channels expressed in HEK 293 cells. (D) Current–voltage relationship for Kv1.1 WT (7 μg; black circle), A401T (7 μg; gray circle), and Kv1.1 WT + A401T (3.5 μg + 3.5 μg; red circle) channels. The inset shows individual current values for the indicated channels at +40 mV (*n* = 21–36). (E) Voltage‐dependent activation curves for the indicated channels. The inset shows individual V_1/2_ values (*n* = 8–17 cells). Kinetics of activation (F) and deactivation (G) measured for the indicated channels. The time constants, resulting from the fit of the activating and deactivating current traces with a single exponential function, were plotted as a function of voltage (*n* = 6–15 cells). (H) Bar graphs showing the fast and slow time constants of the C‐type inactivation for the indicated channels calculated by fitting current decay with a double exponential function (*n* = 12–14 cells). (I) Recovery from inactivation for the indicated channels (*n* = 5–8 cells).

To evaluate whether the reduced current density observed in A401T channels was associated with decreased channel expression, we performed western blot analysis on protein extracts from HEK293 cells expressing Kv1.1 WT, A401T, or WT + A401T channels. As shown in Figure 3, expression of the A401T mutant channel was reduced by 39% compared with WT channels. A similar trend was observed under heterozygous conditions (WT + A401T), in which channel expression was reduced by 24% compared with WT. Although these differences did not reach statistical significance, they suggest that impaired channel expression may contribute to the LOF phenotype of A401T and that this effect is only partially compensated by co‐expression with the WT subunit (Figure 3; Figure S3).

**FIGURE 3:**
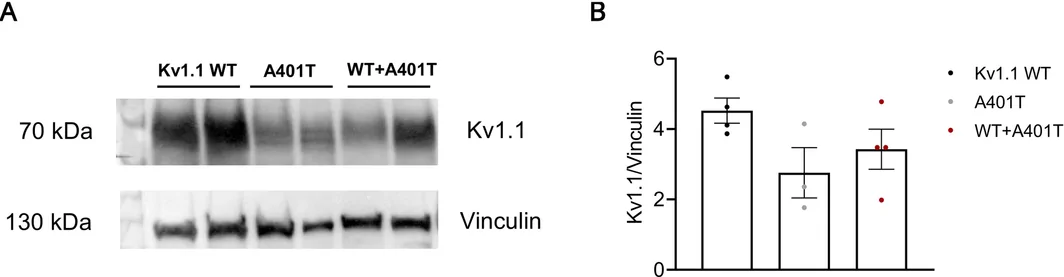
(A) Representative immunoblot showing Kv1.1 protein expression in HEK293 cells transfected with Kv1.1 WT, A401T, or WT + A401T (1:1) constructs. The upper panel shows Kv1.1 immunoreactivity, whereas the lower panel shows vinculin, used as a loading control. (B) Western blot quantification of Kv1.1 protein expression normalized to vinculin in HEK293 cells expressing the indicated channels. Data are mean ± SEM, and individual data points are shown. Sample sizes were: Kv1.1 WT, *n* = 4; A401T, *n* = 3; WT + A401T (1:1), *n* = 4. Kv1.1 expression was normalized to vinculin as a loading control.

## 3 Discussion

Here, we report a *KCNA1* variant identified in a patient with autism and tremor that exhibits a mixed LOF/GOF biophysical profile. In contrast to the closely related Kv1.2 channel, in which GOF and mixed LOF/GOF variants are well recognized, *KCNA1*‐associated neurologic disorders have largely been linked to LOF variants [15]. Mutations affecting the selectivity filter, the conserved PVP motif, or the S4 voltage‐sensing segment are typically associated with severe epileptic phenotypes accompanied by developmental delay and, in some cases, drug resistance [4]. Since 2018, only a few pore‐region *KCNA1* variants have been reported in DEE, several involving the highly conserved PVP motif [1, 20, 21]. To date, no *KCNA1* variant has been associated with autism spectrum disorder. More recently, however, GOF *KCNA1* variants have been described. The de novo A261T variant in the S3 segment, identified in a patient with fever‐induced seizures, ataxia, and myokymia, produced a hyperpolarizing shift in channel activation consistent with a GOF mechanism [7]. Likewise, the S4 variant L296F caused channel activation at more negative potentials in an infant with severe pharmacoresistant epilepsy [8]. These findings suggest that detailed biophysical characterization may help guide precision therapeutic strategies. In contrast, our patient presented with autism and tremor without epilepsy and carried a *KCNA1* variant with a mixed LOF/GOF phenotype.

*KCNA1*‐related disorders may present as autosomal‐dominant familial conditions, particularly in episodic ataxia Type 1, or as apparently sporadic or *de novo* cases [2, 4, 7, 8, 16, 20, 21]. Considerable inter‐ and intrafamilial variability has been reported, and carriers of the same variant may show differences in age at onset, symptom type, and clinical severity, consistent with variable expressivity and incomplete penetrance. In the present family, maternal testing was negative, whereas paternal testing was unavailable because the father was deceased. Consequently, the *de novo* status of A401T cannot be confirmed, and paternal transmission, potentially with reduced penetrance or limited clinical expression, cannot be definitively excluded.

Voltage‐gated potassium channel gating depends on electromechanical coupling between the voltage‐sensing and pore modules [22]. The A401 residue is located within the S6 helix, where it forms a hydrogen bond with Val397 and lies adjacent to the highly conserved PVP motif, a key determinant of S6 flexibility and channel gating (Figure 1A) [23]. Functionally, A401T channels activated at more negative potentials but displayed slower activation kinetics and markedly reduced current amplitude. Replacing alanine with threonine likely perturbs local hydrogen‐bonding interactions and S6 conformational dynamics, impairing transitions between closed and open states (Figure 1A). The reduced current amplitude may result from altered gating, impaired membrane trafficking, or defective subunit assembly. Although not statistically significant, the trend toward reduced protein expression supports a contribution of impaired channel expression or stability to the LOF phenotype, consistent with the patch‐clamp findings. The reduction in current density after co‐expression of WT and A401T subunits further suggests a dominant‐negative effect, although haploinsufficiency cannot be excluded.

The neuronal consequences of a mixed LOF/GOF defect are likely to be circuit‐dependent. Kv1.1/Kv1.2 channels regulate inhibitory transmission at cerebellar basket cell terminals, and their dysfunction disrupts Purkinje cell inhibition, producing ataxia in experimental models [24, 25]. In addition, *Kcna1* knockout mice exhibit increased seizure susceptibility and sudden unexpected death in epilepsy (SUDEP) [26, 27], whereas altered Kv1.1 function in interneurons has also been associated with anxiety‐like behaviors [28]. Together, these findings underscore the complex, context‐dependent effects of Kv1.1 dysfunction.

Several limitations should be acknowledged. Functional studies were performed in a heterologous HEK293 expression system, which does not fully recapitulate neuronal subunit composition or regulatory interactions. In addition, no in vivo or neuronal validation was performed. Complete segregation analysis was not possible because paternal DNA was unavailable; therefore, a *de novo* occurrence cannot be established, and paternal inheritance cannot be excluded. Although whole‐exome sequencing did not identify another potentially relevant variant and no acquired cause of the neurodevelopmental phenotype was identified, the functional effect of A401T does not establish that this variant is the sole cause of the patient's autism spectrum disorder. The contribution of additional genetic, environmental, non‐genetic, or multifactorial factors cannot be excluded, particularly given that this report concerns a single individual.

Overall, our findings expand both the clinical and functional spectrum of *KCNA1*‐related disorders by identifying autism spectrum disorder associated with a mixed LOF/GOF *KCNA1* variant.

## Author Contributions

Juan Darío Ortigoza‐Escobar and Paola Imbrici conceived and organized the work, analyzed and interpreted data; Laura Marti‐Sánchez and Loreto Martorell acquired, analyzed, and interpreted clinical and genetic data; Giorgia Dinoi performed molecular biology experiments and analyzed data; Antonio Vittorio Buono and Antonella Liantonio performed patch clamp experiments and analyzed data; Annamaria De Luca revised the manuscript and interpreted data.

## Funding

This work was supported by the MNESYS FORWARD project “A multiscale integrated platform to promote industrial transition in neuroscience and neuropharmacology”, Code 1122, funded by the Ministry of University and Research (MUR) under Grant Directive No. 767 of 3 March 2026 as part of the National Research, Innovation and Competitiveness Program for the green and digital transition 2021‐2027.

## Ethics Statement

The legal guardians gave their written consent to the recording of the patient for publication, and the study received ethical approval from the Ethics Committee. We confirm that we have read the Journal's position on issues involved in ethical publication and affirm that this work is consistent with those guidelines.

## Conflicts of Interest

The authors declare no conflicts of interest.

## Supporting information


**Figure S1:** Cartoon showing the localization of the Kv1.1 identified variants. Dark blue circles, variants associated with episodic ataxia; magenta circles, variants associated with paroxysmal kinesigenic dyskinesia; yellow circles, variants associated with hypomagnesemia; light blue circles, variants associated with ataxia and epilepsy; green circles, variants associated with musculoskeletal features; purple star, A401T LOF/GOF variant associated with autism and tremor; red circles, variants associated with DEE. The A261T and L296F GOF variants are underlined.
**Figure S2:** Brain magnetic resonance imaging of the patient. Representative coronal (A), midsagittal (B), and axial (C) images obtained at 13 years of age. Cerebral and cerebellar morphology and volume were considered normal on dedicated radiological review, with no evidence of cerebellar atrophy.
**Table S1:** Biophysical parameters of Kv1.1 WT, A401T, and WT + A401T channels expressed in HEK 293 cells.
**Figure S3:** Immunoblots showing Kv1.1 protein expression (upper panels) and the corresponding vinculin controls (lower panels) in protein extracts from untransfected HEK293 cells (CTRL) and HEK293 cells transiently expressing Kv1.1WT, A401T, or WT + A401T (1:1) channels. Kv1.1 immunoreactivity was detected at the expected molecular weight, whereas vinculin was used to verify equal protein loading across samples. Four independent experiments are shown for Kv1.1WT and WT + A401T (1:1), whereas three independent experiments are shown for A401T. Blots were loaded by an operator blinded to the experimental groupings.

## Data Availability

The data that support the findings of this study are available from the corresponding author upon reasonable request.
