# Supplementary material for: A Novel KCNA1 Variant in a Patient With Tremor and Autism Spectrum Disorder Causes Mixed LOF/GOF Defects of Kv1.1 Channels
Source: Ann Clin Transl Neurol. 2026 Sep 21;13(10):2166–71. doi: 10.1002/acn3.70536 (PMC13593927; doi:10.1002/acn3.70536)
Supplement: Supplementary file 1 [file ACN3-13-2166-s001.docx]

Supplementary Material

Materials and Methods

*Genetic analysis*

Whole exome sequencing (WES) was performed using SSel XT HS+XT Low Input Human All Exon V8+NCV (Agilent Technologies) followed by sequencing on an Illumina NovaSeq 6000 platform. Variant annotation and filtering were performed using Alissa Interpret (Agilent Technologies, v5.4.2). The variant was confirmed by PCR amplification of the target region and bidirectional Sanger sequencing using dye-terminator chemistry (BigDye Terminator v3.1) on an ABI capillary sequencer, with alignment to the reference sequence and manual review of electropherograms.

*Mutagenesis and expression of hKv1.1WT and A401T channel*

The A401T mutation was introduced into the plasmid PMT2LF-hKv1.1 containing the full-length human wild-type Kv1.1 cDNA using the QuickChange™ site-directed mutagenesis kit (Stratagene Cloning Systems, Santa Clara, CA, USA)^1^. The complete coding region of the cDNA was sequenced to exclude polymerase errors. HEK293 cells were transiently transfected with the hKv1.1 WT or A401T (3.5 μg or 7 μg) and CD8 reporter plasmids (1 μg) using the calcium–phosphate precipitation method. Only cells binding anti-CD8 antibody-coated microbeads (Dynabeads M450, Invitrogen) were used for patch-clamp recordings.

*Electrophysiology*

Experiments were performed in a heterologous HEK293 expression system. Whole-cell patch-clamp experiments were performed at room temperature (~20 °C) using an Axopatch 200B amplifier and Digidata 1550B AD/DA converter (Axon Instruments, Sunnyvale, CA, USA). Currents were low-pass filtered at 2 kHz and digitized with sampling rates of 50 kHz. The bath solution contained (mM) NaCl, 142; KCl, 2.8; MgCl_2_, 1; CaCl_2_ 1, HEPES, 10; glucose, 11; pH = 7.4, whereas the pipettes were filled with solution containing (mM) NaCl, 10; K-glutamate, 132; MgCl_2_, 2; CaCl_2_, 0.9; EGTA, 1, HEPES, 10, pH = 7.4.^1-3^ The pipettes were pulled from borosilicate glass and had ~4 MΩ resistance. Cells with unstable seal resistance, significant leak currents (>10% of peak current), or series resistance >10 MΩ were excluded from analysis. Protein expression levels and membrane surface trafficking were not directly assessed.

Potassium currents were evoked by 200 ms depolarizing commands from a holding potential of –80 to +60 mV (10 mV intervals). To measure tail currents, each prepulse was followed by a 150 ms voltage step at −50 mV for Kv1.1 wild-type and A401T channels. Normalized tail currents were plotted as a function of the prepulse potential, and data points fitted with the Boltzmann function I = 1/{1 + exp[−(V−V_1/2_)/k]} to determine the voltage dependence of channel activation. V_1/2_, the half-maximal activation potential, and k, the slope factor, were calculated from the fit.

To measure the kinetics of activation, currents were elicited by 200 ms depolarizing pulses from a holding potential of –80 to +60 mV (10 mV intervals). To measure the kinetics of deactivation, currents were elicited by 200 ms depolarizing pulse at +20 mV, followed by 200 ms depolarizing pulses from −80 to +20 mV (10 mV intervals). The kinetics of activation and deactivation were determined by fitting activating and deactivating current traces with a single exponential function. The calculated time constants were plotted as a function of voltage and fitted with the equation τ = τ_V1/2_ exp(V−V_1/2_)/k, where τ_V1/2_ is the time constant at the V_1/2_ of the channels, and k is the slope factor for the voltage dependence of the time constants.

To determine the slow C-type inactivation, a test pulse to +40 mV was delivered for 90 s to cells expressing wild-type and mutant channels. The kinetics of inactivation were estimated by fitting the time course of current decay with a double exponential function and calculating the fast (τ_fast_) and slow (τ_slow_) time constants.

The recovery from the C-type inactivation was determined by using a double-pulse protocol to +40 mV, separated by interpulse intervals of increasing duration (from 0.1 to 17.1 s). The peak currents evoked by the second pulse (100 ms) were divided by the first pulse (20 s), normalized, and plotted as a function of the interpulse duration. Data points were fitted with a single exponential function from which the time constants were calculated^1–3^.

Data were analyzed by using pCLAMP 10.3 (Axon Instruments, Sunnyvale, CA, USA) and GraphPad Software.

*Western blot analysis*

Total protein extracts were obtained from HEK293 cells transiently transfected using the calcium phosphate (CaCl₂) precipitation method with different plasmid combinations: WT (7 μg), A401T (7 μg), and WT+A401T (3.5 μg + 3.5 μg). Untransfected cells were used as negative controls. All experiments were performed in 3-4 independent biological replicates.

48 hours after transfection, cells were lysed using RIPA buffer^4^. Total protein concentration was quantified using the bicinchoninic acid (BCA) Protein Assay Kit (Sigma-Aldrich, USA; bicinchoninic acid solution, cat# 1003278214; copper sulfate solution, cat# 1003274018), according to the manufacturer’s instructions. A total of 10 μg of protein per sample was separated on 10% Mini-PROTEAN TGX Stain-Free Protein Gels (Bio-Rad, Hercules, CA, USA; cat# 4568034) and transferred onto stain-free polyvinylidene difluoride (PVDF) membranes (Bio-Rad, Hercules, CA, USA; cat# 1704156) using the Trans-Blot® Turbo™ Transfer System (Bio-Rad, Hercules, CA, USA) for 7 min at 25 V and 1.3 A.

Membranes were blocked for 1 h at room temperature in 5% non-fat dry milk blocking solution (Bio-Rad, Hercules, CA, USA) and subsequently incubated overnight at 4 °C with primary antibodies against Kv1.1 (1:1000 dilution; protein tech, cat# 68008-1-Ig, RRID:AB_2918755). Successively, membranes were washed three times for 10 min each with Tris-Buffered Saline with Tween 20 (TBST buffer) and subsequently incubated with secondary anti-mouse antibody (1:5000 Goat anti-Mouse IgG Fc Secondary Antibody, HRP, RRID:AB_2534758 horseradish peroxidase-conjugated secondary antibodies) at room temperature for 1h. Protein bands were developed with a chemiluminescent substrate (Clarity Western ECL Substrate, Bio-Rad, Hercules, CA, USA, cat# 1705061) and signals were recorded with a Chemidoc MP imaging system (Bio-Rad, Hercules, CA, USA). Densitometric analysis of each protein band was performed as detailed in^5^, using anti-vinculin as reference protein (dilution 1:1000, Santa Cruz Biotechnology Cat# sc-73614 HRP, RRID:AB_2941767).

*Statistics*

Results were reported as mean ± standard error of mean (SEM) from n cells. Statistical comparisons between WT, A401T and WT+A401T were performed using one-way analysis of variance (ANOVA) followed by Tukey's multiple comparisons post-hoc test, using the GraphPad Prism version 8 program. P < 0.05 was considered statistically significant. Sample sizes were consistent with previous electrophysiological studies of Kv1.1 variants and were not determined by formal power calculation. Data acquisition and analysis were not performed blinded to experimental condition.

**Supplementary Figure 1**. Cartoon showing the localization of the Kv1.1 identified variants. Dark blue circles, variants associated with episodic ataxia; magenta circles, variants associated with paroxysmal kinesigenic dyskinesia; yellow circles, variants associated with hypomagnesemia; light blue circles, variants associated with ataxia and epilepsy; green circles, variants associated with musculoskeletal features; purple star, A401T LOF/GOF variant associated with autism and tremor; red circles, variants associated with DEE. The A261T and L296F GOF variants are underlined.

**Supplementary Figure 2.** Brain magnetic resonance imaging of the patient. Representative coronal (A), midsagittal (B), and axial (C) images obtained at 13 years of age. Cerebral and cerebellar morphology and volume were considered normal on dedicated radiological review, with no evidence of cerebellar atrophy.

**Supplementary Table 1.** Biophysical parameters of Kv1.1 WT, A401T, and WT+A401T channels expressed in HEK 293 cells.

|  | **Current +40mV** | **Voltage Dependence**  **of Activation** | | **Kinetic of**  **Activation** | **Kinetic of**  **Deactivation** | **C-type Inactivation**  **+40 mV** | | **Recovery from Inactivation** |
| --- | --- | --- | --- | --- | --- | --- | --- | --- |
|  | (nA) | V_1/2_ (mV) | k (mV) | τ_V1/2_ (ms) | τ_V1/2_ (ms) | τ_fast_ (s) | τ_slow_ (s) | τ (s) |
| **Kv1.1 WT** | 2.10±0.20  (26) | −26.9±1.5  (15) | 8.1±0.6  (15) | 20.4±1.1  (15) | 42.8±2.9  (14) | 6.2±0.4  (13) | 38.6±2.2  (13) | 4.7±0.6  (8) |
| **A401T** | 0.27±0.02*  (21) | -39.5±1.2*  (8) | 4.2±0.4*  (8) | 30.4±2.6*  (8) | 40.3±0.6  (6) | 5.3±0.6  (12) | 25.0±1.3*  (12) | 3.1±0.5  (5) |
| **WT + A401T** | 0.84±0.13°  (36) | −41.4±2.4°  (17) | 7.9±0.5^#^  (17) | 16.0±1.8^#^  (11) | 33.9±1.1  (12) | 4.9±0.5  (14) | 29.1±1.7°  (14) | 5.4±1.2  (7) |

Data are shown as mean ± SEM. The number of cells is indicated in brackets.

Statistics: one-way ANOVA (F=34.95, p<0.0001 for current; F=15.98, p<0.0001 for V_1/2_ of voltage dependence of activation; F=11.71, p<0.0001 for k value; F=15.41, p<0.0001 for τ_V1/2_ of kinetics of activation; F=15.41, p<0.0001 for τ_slow_ of C-type inactivation). Tukey's multiple comparisons post-hoc test was as follows: * WT vs A401T (0.0001<p<0.0015); ° WT vs WT+A401T (0.0001<p<0.0015); ^#^ A401T vs WT+A401T (0.0001<p<0.0003).

**Supplementary Figure 3.** Immunoblots showing Kv1.1 protein expression (upper panels) and the corresponding vinculin controls (lower panels) in protein extracts from untransfected HEK293 cells (CTRL) and HEK293 cells transiently expressing Kv1.1WT, A401T, or WT+A401T (1:1) channels. Kv1.1 immunoreactivity was detected at the expected molecular weight, whereas vinculin was used to verify equal protein loading across samples. Four independent experiments are shown for Kv1.1WT and WT+A401T (1:1) whereas three independent experiments are shown for A401T. Blots were loaded by an operator blinded to the experimental groupings.

**References**

1.	Dinoi G, Morin M, Conte E, et al. Clinical and Functional Study of a De Novo Variant in the PVP Motif of Kv1.1 Channel Associated with Epilepsy, Developmental Delay and Ataxia. Int J Mol Sci 2022;23(15). https://doi.org/10.3390/ijms23158079

2.	Imbrici P, Gualandi F, D’Adamo MC, et al. A novel KCNA1 mutation identified in an Italian family affected by episodic ataxia type 1. Neuroscience 2008;157(3):577–587. <https://doi.org/10.1016/j.neuroscience.2008.09.022>

3.	Imbrici P, Cusimano A, D’Adamo MC, De Curtis A, Pessia M. Functional characterization of an episodic ataxia type-1 mutation occurring in the S1 segment of hKv1.1 channels. In: Pflugers Archiv European Journal of Physiology. Springer Verlag, 2003;p373–379. https://doi.org/10.1007/s00424-002-0962-2

4.	Altamura C, Conte E, Campanale C, Laghetti P, Saltarella I, Camerino GM, Imbrici P, Desaphy JF. Chaperone activity of niflumic acid on ClC-1 chloride channel mutants causing myotonia congenita. Front Pharmacol. 2022 Aug 11;13:958196. doi: 10.3389/fphar.2022.958196

5.	Conte E, Imbrici P, Dinoi G, Boccanegra B, Lanza M, Mele E, Riemma MA, Urbanek K, Cappetta D, De Luca A, Berrino L, De Angelis A, Liantonio A. SGLT2 inhibitor dapagliflozin mitigates skeletal muscle pathology by modulating key proteins involved in glucose and ion homeostasis in an animal model of heart failure. Eur J Pharmacol. 2025 Jun 15;997:177617. doi: 10.1016/j.ejphar.2025.177617
